# Lower Intakes of Key Nutrients Are Associated with More School and Workplace Absenteeism in US Children and Adults: A Cross-Sectional Study of NHANES 2003–2008

**DOI:** 10.3390/nu15204356

**Published:** 2023-10-13

**Authors:** Qian Ye, Prasad P. Devarshi, Ryan W. Grant, Kelly A. Higgins, Susan H. Mitmesser

**Affiliations:** 1 Science & Technology, Pharmavite LLC, West Hills, CA 91304, USA; pdevarshi@pharmavite.com (P.P.D.); rgrant@pharmavite.com (R.W.G.);; 2 Food Components and Health Laboratory, Beltsville Human Nutrition Research Center, Agricultural Research Service, US Department of Agriculture, Beltsville, MD 20705, USA

**Keywords:** absenteeism, supplements, nutritional status, dietary intake, children, adults, school, workplace, NHANES

## Abstract

The influence of individual macro- and micronutrients on absenteeism in the United States is largely unknown. The objective of this study was to determine whether nutritional status or nutrient intake were associated with absenteeism from school and work due to illness or injury. Data from NHANES 2003–2008 were used to assess nutrient intake from food and food plus supplements, nutritional biomarker levels, and school and work absenteeism per year in children and adults. Negative binomial regression models were used to predict mean days of missed work per year and to estimate incidence rate ratios (IRRs) of absenteeism by nutrient biomarker status. Of 7429 children, 77% reported missing school days (mean 4.0 days). Of 8252 adults, 51% reported missing work days (mean 4.9 days). Children and adults who reported more absent days had a significantly lower intake of protein and several essential micronutrients from the diet. When nutrients from supplements were included, this negative association was retained for protein, selenium, choline, and DHA in children and for protein, selenium, vitamin K, choline, potassium, fiber, octadecatrienoic acid, and lycopene in adults. Future studies are needed to ascertain whether dietary interventions, such as access to healthier food options and/or dietary supplements, can reduce absenteeism.

## 1. Introduction

Absenteeism from school and work can have long-term negative consequences. School absenteeism in children can lead to reduced academic achievement and poor socioemotional outcomes [1,2,3]. School absenteeism in earlier years is linked to more absenteeism in later school years [3]. Workplace absenteeism in adults can result in productivity loss, which leads to substantial costs to employers [4,5]. Absenteeism is an outcome we should pay attention to as it is linked to higher dropout rates, lower rates of high school graduation and college enrollment, lower earned income, and poorer health [6]. Contributors to absenteeism in children and adults include poor physical health [7,8,9] and illness [6]. Physical health, such as lack of participation in physical activity [10,11] and elevated body mass index (BMI) [12,13], have also been independently linked to missed days of school and work.

Physical health is heavily influenced by nutritional status. However, the relationships between diet, particularly individual nutrients, and absenteeism are largely unknown. Studies monitoring nutritional status and absenteeism focus mostly on the implementation of school feeding programs or workplace wellness programs [14,15,16,17,18]. But these studies usually combined dietary programs with other interventions [18], and absenteeism, if measured at all, was often a secondary outcome [16,17,18]. Workplace programs were usually limited to educational sessions [16], which means they only encourage a portion of healthy food to be added to the total diet, but do not impose long-term dietary change.

Very few studies have assessed the association between individual nutrients on school and work absenteeism in the US. Previous studies with children have shown an association between increased absenteeism and food insecurity [19]. Assessment of macronutrient composition is limited to protein intake among children in resource-limited countries; in these studies, school feeding programs resulted in higher protein intake and lower absenteeism rates among participants living in Kenya [20] and Peru [21]. In a sample of schoolchildren in Colombia, those with vitamin B12 deficiency had a higher adjusted absenteeism rate [22]. Among adults, decreased absenteeism from work has been linked to a self-reported “healthy” diet [8] and adherence to a higher-quality diet (i.e., Dietary Approaches to Stop Hypertension [DASH] diet) [23]; however, macro- and micronutrient intakes were not assessed in these studies. High fruit and vegetable consumption, which is associated with high micronutrient intake [as reviewed by Fulton 2016 [24]], has been associated with reduced absenteeism from work [8]. 

While the literature supports the importance of nutrition in physical health, the role of nutritional status, on school and work absenteeism in children and adults in the US is largely unknown. Additionally, combining self-report assessments of dietary intake with objective measurements of nutrient biomarkers can strengthen the validity of study results. Given the popular use of supplements in the US, which are consumed by approximately 30% of US children [25] and 80% of US adults [26], it is important to consider the contribution of supplements when examining the role of micronutrients in school and work absenteeism. Therefore, the objective of this study was to determine whether nutritional status or nutrient intake (including food and supplement intake), as assessed with 24 h dietary recalls and nutrient biomarkers, are associated with absenteeism in school and work using cross-sectional data that is nationally representative of the US population.

## 2. Materials and Methods

### 2.1. Database and Study Population

Data were used from 3 cycles of the National Health and Nutrition Examination Survey (NHANES), 2003–2004 [27], 2005–2006 [28], and 2007–2008 [29], a cross-sectional examination of a nationally representative sample of children and adults in the US. NHANES uses a multistage probability sampling design to continuously survey a sample representative of the civilian, noninstitutionalized household population in the US. Multiple sample persons could be sampled from the same household (on average 2 sample persons per household) [30]. NHANES data collection includes a household interview, an interview and health examination at a mobile examination center (MEC), and post-MEC follow-up data collection over the phone. The most recent cycles of NHANES do not collect absenteeism data; therefore, the analysis as restricted to NHANES 2003–2008, the most recent cycles of the survey that capture information on work and school absenteeism. All NHANES protocols were approved by the National Center for Health Statistics (NCHS) Research Ethics Review Board and underwent annual review [31]. All sample persons were informed of the study procedures and provided consent to complete the home interview and the health examination. Sample persons at age of maturity provided consent to participate in the survey. Parents or guardians of sample persons ≤ 17 years (y) of age gave permission for minors to participate in the survey; sample persons 7–17 y of age also provided assent to participate.

**Figure 1:**
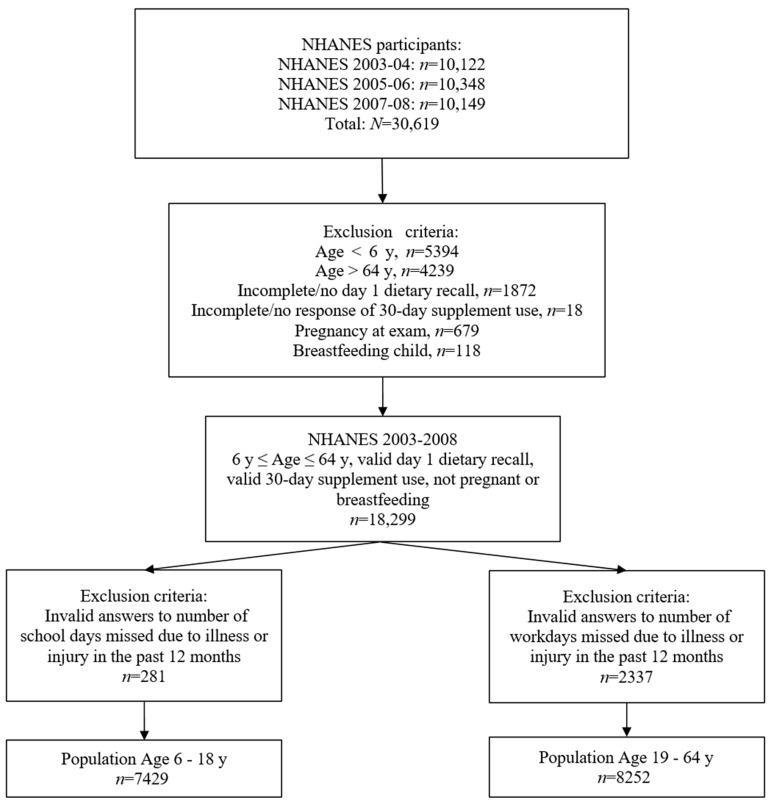
Population included in analysis, NHANES 2003–2008. NHANES, National Health and Nutrition Examination Survey; y, year(s).

The target populations for this analysis consisted of children 6 to 18 y of age for absenteeism in school, and adults 19 to 64 y of age for absenteeism in the workplace. The population was restricted to individuals with valid day 1 dietary recalls, 30-day supplement questionnaires, and answers to the number of school or work days missed; pregnant and lactating women were excluded (Figure 1). 

### 2.2. Absenteeism Data

Data on absenteeism from school and work were collected as part of the Medical Conditions Questionnaire (MCQ) during the 2003–2008 NHANES cycles using the Computer-Assisted Personal Interviewing-CAPI (interviewer administered) system. For the 6 to 18 y subpopulation, the number of school days missed from injury/illness was obtained from item MCQ150Q, which stated, “During the past 12 months, about how many days did you miss school because of an illness or injury?”. For the 19 to 64 y subpopulation, the number of work days missed was obtained from item MCQ245B, which stated, “During the past 12 months, about how many days did you miss work at a job or business because of an illness or injury (do not include maternity leave)?”. Sample persons ≥ 16 y provided responses to the MCQ while a responsible adult provided responses for sample persons < 16 y [32,33,34]. A responsible adult also provided responses for sample persons who could not self-report [32,33,34].

### 2.3. Dietary Intake Data

Dietary intake data were collected during the MEC interview as part of the What We Eat in America (WWEIA) component of NHANES. Trained dietary interviewers completed a dietary recall to collect detailed information on all food and beverages consumed by respondents in the previous 24 h time period (midnight to midnight) using the United States Department of Agriculture (USDA) Automated Multiple-Pass Method in either English or Spanish [35]. A second dietary recall was collected by telephone 3 to 10 days after the first dietary interview, but not on the same day of the week as the first interview. Dietary data was self-reported among participants ≥ 12 y and was self-reported with assistance from an adult household member among participants 6–11 y [36,37,38,39,40,41]. In select circumstances when the sample person and the proxy cannot provide sufficient dietary data, NHANES implemented data retrieval procedures to obtain dietary data from someone outside of the household [36,38,40]. 

Estimated nutrient intakes from the diet were calculated using survey-specific USDA Food and Nutrient Database for Dietary Studies. Estimated nutrient intake from supplements was based on the 30-day supplement intake data that were collected during the household interview. NHANES had not processed the intake from food for total choline (for 2003–2004) or vitamin D (for 2003–2004 and 2005–2006); therefore, the dietary intake analyses were restricted to the NHANES 2005–2006 and 2007–2008 cycles for total choline and to the NHANES 2007–2008 cycle for vitamin D. Also, the total nutrient intake from the 30-day supplement use questionnaire was processed only for the 2007–2008 cycle. Therefore, the unprocessed 30-day supplement use data and the Dietary Supplement Product Information and Dietary Supplement Ingredient Information databases were used to estimate nutrient intake from supplement use for NHANES 2003–2004 and 2005–2006 participants prior to running the analyses. The same process was used to process intake of vitamin A, alpha-carotene, beta-carotene, beta-cryptoxanthin, vitamin E, octadecatrienoic acid (polyunsaturated fatty acid [PUFA] 18:3), eicosapentaenoic acid (EPA; PUFA 20:5), and docosahexaenoic acid (DHA; PUFA 22:6) for all cycles, which were not provided in NHANES.

### 2.4. Nutrient Biomarkers

Blood and urine specimens were collected at the MEC and assessed for analyte levels. Clinically relevant cutoffs have been established for vitamin A [42,43], vitamin C [43], vitamin D [44], vitamin E [43], vitamin B6 (pyridoxal-5′-phosphate [PLP] and 4-pyridoxic acid [4-PA] [45], vitamin B12 (based on the combined analysis of serum B12 and methylmalonic acid; [43,46,47]), folate (serum and red blood cell [RBC]; [45]), iron (serum ferritin and soluble transferrin receptor [sTfR]; [48,49,50]), and urinary iodine [51,52]. Serum ferritin was only measured among females aged 12 to 49 y; therefore, serum ferritin was evaluated in these subgroups only (12–18 y, 19–49 y) [53]. As a joint analysis of serum vitamin B12 and MMA may be a more sensitive and specific tool to determine vitamin B12 deficiency at the population level, joint vitamin B12 and MMA levels were used to assess vitamin B12 status, as demonstrated in Bailey et al. [47].

Recognized cutoffs were used to categorize participants by nutrient status if the number of participants per category was 100 or more; participants in each group were classified by tertiles of biomarker distribution levels if the number of participants in a given category was less than 100 or recognized biomarker cutoffs were not available.

### 2.5. Statistical Analysis

All statistical analyses were performed using STATA^®^ (Version 12.1, StataCorp LLC, College Station, TX, USA) and SAS^®^ software (Version 9.4, SAS Institute Inc., Cary, NC, USA). All analyses used the statistical weights provided by NCHS, and standard errors and statistical tests were adjusted for survey design. Statistical significance was set at *p* ≤ 0.05 for all analyses. 

Negative binomial regression models were used to predict number of missed school and work days per year for each age group (6 to 18 y and 19 to 64 y). Participants were classified into 2 subcategories: lower absenteeism and higher absenteeism. Participants were classified as lower absenteeism if the number of missed school or work days was less than or equal to the predicted mean number of days missed. Participants were classified as higher absenteeism if the number of missed school or work days was more than the predicted mean number of days missed.

Mean intakes were estimated from micro- and macronutrient intake data from food and supplements for both age groups. For each nutrient of interest, usual intakes from food only and from food + supplements were derived using the National Cancer Institute (NCI) method and the SAS^®^ macros developed by NCI for the modeling of a single dietary component [54]. Usual nutrient intake analyses were adjusted for day of recall, recall day of the week (weekday/weekend), dietary reference intake (DRI) age group, gender, and dietary supplement use.

Usual intake estimates from food alone and food + supplements for lower absenteeism and higher absenteeism groups were generated to determine the association between nutrient intake and absenteeism. Models were adjusted for day of recall, recall day of the week (weekday/weekend), DRI age group, BMI, gender, race/ethnicity, household income, education (for workplace absenteeism) or head of household (HH) education (for school absenteeism), marital status (for workplace absenteeism) or HH marital status (for school absenteeism), and dietary supplement use. Unadjusted models are available upon request. Mean usual nutrient intake from food alone and food + supplements for the lower absenteeism and higher absenteeism groups were compared by biomarker category using a z test. Methods similar to those used by Andreyeva et al. [12] were used to analyze the association between absenteeism and nutrient biomarkers. Specifically, participants were classified into biomarker categories based on clinically relevant cutpoints (i.e., nutrient sufficient, deficient based on recognized biomarker cutoffs) or tertiles of biomarker distribution levels. Negative binomial regression models were used to estimate the incidence rate ratios (IRRs) of absenteeism and missed school and work days by biomarker status category, adjusting for the aforementioned covariates. Analyses were run separately for the two age groups. 

## 3. Results

### 3.1. Population Characteristics

Most participants in the 6 to 18 y group were non-Hispanic white (62.5%), with a normal BMI (63.1%), and did not use dietary supplements (70.6%; Table 1). Most participants in the 19 to 64 y group were non-Hispanic white (70.9%; Table 1). The distribution of BMI categories was relatively equal between normal weight (32.7%), overweight (33.1%), and obese (32.6%) among adults. Dietary supplements were consumed by relatively half of the adult population (48.4%). 

In the 6 to 18 y age group, 77% reported missing school days due to illness/injury, with an average of 5.20 days missed (Table 2). The predicted mean number of school days missed was 3.99 days; most participants (64%) missed less than or equal to this predicted number of days. In the 19 to 64 y age group, 51% reported missing work days due to illness/injury, with an average of 9.60 days missed. The predicted mean number of work days missed was 4.79 days; most participants (84%) missed less than or equal to this predicted number of days. The predicted mean missed school or work days and absolute mean missed school or work days were similar among both age groups.

### 3.2. Nutrient Intake

Usual intake levels from food alone and food + supplements for children 6 to 18 y are provided in the supplementary material (Table S1). Children ages 6 to 18 y in the higher absenteeism group (i.e., missed school days more than the predicted mean number of days) consumed lower levels of protein, vitamin B6, folate, vitamin B12, iron, zinc, selenium, total choline, beta-cryptoxanthin, and DHA (PUFA 22:6) from food alone than those in the lower absenteeism group (i.e., missed school days less than or equal to the predicted mean number of days; Table 3). 

When nutrients from supplements were included, children ages 6 to 18 y who reported more absent days consumed less protein, selenium, total choline, beta-cryptoxanthin, and DHA from food + supplements than those who reported less absenteeism (Table 3). 

Usual intake levels from food alone and food + supplements for adults 19 to 64 y are provided in the supplementary material (Table S2). Adults ages 19 to 64 y in the higher absenteeism group consumed less protein, vitamin E, vitamin B1 (thiamin), vitamin B3 (niacin), vitamin B6, folate, vitamin C, selenium, vitamin K, total choline, potassium, dietary fiber, octadecatrienoic acid (PUFA 18:3), alpha-carotene, and lycopene from food alone than those in the lower absenteeism group (Table 4). When nutrients from supplements were included, adults ages 19 to 64 y in the higher absenteeism group consumed less protein, selenium, vitamin K, total choline, potassium, dietary fiber, octadecatrienoic acid (PUFA 18:3), alpha-carotene, beta-carotene, lycopene, lutein + zeaxanthin, total PUFAs, EPA (PUFA 20:5), and DHA (PUFA 22:6) than those who reported less absenteeism (Table 4).

### 3.3. Nutrient Biomarkers

Distribution of the study population based on clinically relevant biomarker cutpoints and by tertiles are provided in the supplementary material (Tables S3 and S4). For female children ages 12 to 18 y, higher IRRs of absenteeism and more school absent days per year were observed for females with depleted iron stores (i.e., serum ferritin levels < 15 μg) compared to females with serum ferritin levels in the normal range (15 to 150 μg/L) (IRR = 1.36, *p* = 0.02; mean additional absent days = 1.13, *p* = 0.03) (Table S5). However, no effect was observed for other biomarkers of iron status. Higher IRRs of absenteeism and more school absent days per year were observed for the lowest tertile of serum total folate compared to the highest tertile (IRR = 1.16, *p* = 0.05; mean additional absent days = 0.57, *p* = 0.04). Compared with the highest tertile of B12, higher IRRs of absenteeism and more school absent days per year were observed for the lowest tertile of B12 (IRR = 1.29, *p* = 0.01; mean additional absent days = 0.94, *p* = 0.01) and middle tertile of B12 (IRR = 1.2, *p* = 0.02; mean additional absent days = 0.65, *p* = 0.02). However, this effect was not observed in the joint B12 and MMA analysis. No other associations were observed between the other biomarkers and absenteeism.

In adults ages 19 to 64 y, a higher IRR of absenteeism and more work absent days per year were observed for the group deficient or potentially deficient in B12, based on B12 and MMA levels compared with the group that was adequate or normal B12 (IRR = 2.32, *p* = 0.003; mean additional days absent = 6.7, *p* = 0.01) (Table S6). Additionally, a higher IRR of absenteeism and more work absent days per year were observed for the lowest tertile of B12 compared to the highest tertile group (IRR = 1.41, *p* = 0.01; mean additional days absent = 1.6, *p* = 0.01). A lower IRR and less absenteeism days per year were observed for the middle tertile group of B6 (4-PA) compared to the highest tertile group (IRR = 0.77, *p* = 0.03; mean additional days absent = −1.11, *p* = 0.03), but the difference between the lowest and the highest tertile was not statistically significant. In females 19 to 49 y (the only adult population in which iron biomarkers were collected), a lower IRR and less absenteeism days per year were observed in participants with depleted iron stores compared to participants in the non-depleted category (IRR = 0.59, *p* = 0.02; mean additional days absent = −1.99, *p* = 0.01). No other associations were observed between the other biomarkers and absenteeism.

## 4. Discussion

The purpose of this study was to determine if nutritional status or nutrient intake were associated with absenteeism in school and work. Our findings suggest that an intake of protein and several micronutrients and levels of several nutrient biomarkers were associated with increased absenteeism in children and adults. 

### 4.1. Absenteeism and Macronutrient Status

In the current study, less protein intake was associated with increased absenteeism in children, and less protein and dietary fiber intake were associated with increased absenteeism in adults. While participation in school feeding programs has been associated with increased protein intake and reduced absenteeism in resource-limited countries [20,21], the relationship between protein intake and absenteeism from school and work has not been examined in the US. Similarly, only one study has directly assessed fiber and absenteeism: in 219 children ages 3 to 6 y, there was no effect of fiber supplementation on absence from kindergarten [55]. No studies have directly examined fiber intake and absenteeism from work in adults. Future studies are needed to determine the role of protein, fiber, and other macronutrients in absenteeism in US populations. 

### 4.2. Absenteeism and Micronutrient Status

Intake of several B vitamins, including vitamins B1 (thiamin), B3 (niacin), B6, B12, and folate and choline, were significantly associated with absenteeism in children and/or adults in this study. B vitamins, in particular folate and B12, are critical to one-carbon (1C) metabolism, a set of biochemical pathways that regulate methylation reactions involved in DNA synthesis, amino acid homeostasis, antioxidant generation, and epigenetic regulation [56,57]. As such, B vitamins may influence absenteeism through their role in physical health and disease pathology. Although B vitamin deficiencies were not observed for the majority of individuals in this study, their intake of B vitamins may not be sufficient for “optimal” functioning. This could affect their health through minor illnesses or infections that could increase absenteeism. Indeed, vitamin B12 specifically may contribute to the immune response by increasing the number of CD8+ T cells and natural killer T-cell activity, thus improving immune function [58]. Folate has also been implicated in numerous immune functions [59], and deficiencies can result in decreased resistance to infections [60]. B vitamins have also been implicated in mental health and may contribute to attitudes or feelings about school and work. Negative associations between depression severity and vitamin B12 [61,62], B6 [63], and choline [64] levels have been reported, which may affect an individual’s ability or desire to attend school or work. Furthermore, higher serum folate concentrations were associated with higher cognitive test scores in children in a previous analysis of NHANES data [65]. Children who perform better in school may be more likely to attend and miss less days (and vice versa). More studies are needed to determine potential mediators of the relationship between B vitamins and absenteeism. 

In this study, an intake of select vitamins (vitamin C and vitamin E) and minerals (zinc, selenium) that provide antioxidant support were significantly associated with absenteeism in children and/or adults. Antioxidants are essential for an effective immune response against infectious pathogens, and dietary deficiencies can impair immunity and lead to increased infections (as reviewed by Puertollano et al. [66]). Although the effects of vitamin C and zinc on preventing the common cold and/or an upper respiratory tract infection were mixed, it has been consistently shown that vitamin C [67,68] and zinc [68,69,70,71] supplementation can help to shorten the duration of illness. This may explain why a higher intake of these nutrients is associated with lower absenteeism. More research is needed to determine if antioxidant supplementation, either alone or in combination with other vitamins (e.g., B vitamins), reduces absenteeism in children and adults living in the US. 

For children, there were three nutrients that were significantly inversely associated with absenteeism in both dietary intake (food alone) and biomarker status in this study: vitamin B12, serum total folate, and iron (serum ferritin). However, associations were not observed for all markers of vitamin B12 (joint vitamin B12 and MMA), folate (RBC folate), and iron status (serum transferrin receptor, transferrin saturation, and total body iron). Serum folate is a better measure of short-term folate status, whereas erythrocyte folate is a better indicator of long-term folate status [72]. The lack of association between RBC folate and absenteeism is consistent with results from previous results among Colombian children [22]. Consistent with the current analysis, Colombian children with plasma vitamin B12 levels < 148 pmol/L had a 1.89-times higher adjusted rate of school absenteeism compared with participants with levels ≥ 148 pmol/L [22]. However, the Duong et al. study did not observe an association between serum ferritin and absenteeism. The children included in the study were younger (5 to 12 y), included both males and females, and were Colombian. These factors may have influenced the differences observed in the results between the two studies. 

For adults, vitamin B12 deficiency or potential deficiency (as assessed by joint vitamin B12 and MMA levels) were associated with more absenteeism from work in our study. This relationship has not been studied previously, and more studies are needed to confirm these results. Interestingly, among females aged 19 to 49 y, lower absenteeism was observed in this study for individuals with depleted iron stores compared to individuals in the non-depleted category. This is contradictory to our hypothesis that nutrient-deficient populations would have higher absenteeism. Serum ferritin is a biomarker sensitive to iron deficiency, but this biomarker does not indicate the level of severity of deficiency [73]. Furthermore, this relationship may be confounded by inflammation. Ferritin is a positive acute-phase protein that increases during inflammation in addition to its role in iron storage [50]. There are currently no recognized methods to account for inflammation when interpreting ferritin levels. Given the lack of significant associations between absenteeism and the other markers for iron status, the association with ferritin should be interpreted with caution. 

In adults, lower IRR and less absenteeism days were observed in this study for the middle tertile of 4-PA compared to the highest tertile. While 4-PA is a metabolite of vitamin B6, 4-PA values are a poor indicator of vitamin B6 status because levels are heavily influenced by recent intake and renal function status [47,74]. While a combination of functional and direct biomarkers to assess vitamin B6 status is recommended [74], these biomarkers were not measured in NHANES. Serum PLP, the active coenzyme form of vitamin B6, is generally viewed as the best single indicator of vitamin B6 status because it reflects both dietary intake and tissue stores, and PLP changes slowly in response to changes in dietary intake [75]. In our study, adults with lower PLP values had a higher IRR with a trending *p*-value of 0.09 and 2 more absent days (*p* = 0.13) compared with adults with higher PLP values. The lack of significant association with absenteeism in this study may be due to the use of a single biomarker instead of a combination, as is recommended. 

### 4.3. Absenteeism and Supplement Intake

Lower usual nutrient intakes were significantly associated with higher absenteeism for certain nutrients when analyses were conducted based on food only, but not food + supplements. These nutrients, including vitamins C, E, B1 (thiamine), B3 (niacin), B6, and folate in adults, and vitamins B6, B12, folate, iron, and zinc in children, can be commonly found in supplements. A possible explanation for this relationship is that individuals with higher absenteeism may seek out the use of dietary supplements as a means to improve their health [76,77]. Interestingly, lower usual intakes of beta-carotene, lutein + zeaxanthin, total PUFAs, EPA, and DHA were significantly associated with higher workplace absenteeism in adults in the food + supplements analyses. This may indicate that among supplement consumers, a negative association exists. The data need to be interpreted with caution because (1) the percentages of participants that consumed these nutrients from supplements was low (2.6% to 27.2% of adults, depending on the nutrient; data available upon request); and (2) intakes from supplements may not contribute substantially to the total usual nutrient intake because the mean intakes of these nutrients were very similar from food alone and food + supplements. To fully understand the association of supplemental intake of beta-carotene, lutein + zeaxanthin, total PUFAs, EPA, and DHA and absenteeism, oversampling of people who take supplements with these nutrients may be needed. However, whether the use of dietary supplements causally improves absenteeism cannot be determined with this cross-sectional data. Future studies are needed to ascertain whether higher nutrient intake from food and supplements, particularly for nutrients not commonly found in the typical American diet, is an effective intervention to reduce absenteeism.

### 4.4. Limitations

Limitations of the current study include the cross-sectional study design, which cannot establish any cause-and-effect relationships between nutritional status and absenteeism. Analysis on more recent data was not possible because absenteeism data was not collected in later cycles of NHANES. Dietary intake, supplement use, and diet quality have changed over time [78,79,80], which could result in altered incidence, severity, and causes of absenteeism, highlighting the importance of capturing this data in future cycles of NHANES. In addition, the reasons for absenteeism measured in NHANES include both illness and injury, and injury may be caused by non-health related issues (e.g., motor vehicle accidents). Furthermore, the absenteeism and dietary and supplement intake data were self-reported outcomes, which may be subject to recall bias and not accurately reflect actual days missed or nutrients consumed. The USDA Automated Multi-Pass Method is a validated method to measure energy and nutrient intake among adults [35,81], but limited research has been conducted to assess the method’s validity among children. There are challenges when collecting self-reported dietary data among children due to limited literacy and writing skills, food recognition skills, memory, and concentration [82]; in addition, children often play a limited or no role in preparing the foods they consume. While an adult household member assisted in providing dietary data among participants 6–11 y in this survey, these adults may be limited in their ability to provide detailed information on the foods prepared by other care givers or at school. In select circumstances when the sample person and the proxy cannot provide sufficient data, NHANES implemented data retrieval procedures to obtain dietary data from someone outside of the household [36,38,40]. Despite the limitations of self-reported dietary data, it still provides valuable information about dietary intake among both adults, adolescents, and children [83]. 

Biomarker assessment of nutrient levels was included to provide a more objective measure of nutrient consumption. However, limitations also exist for biomarker measures: recognized cutoffs may not exist for certain biomarkers, and biomarkers can be affected by other biological processes and nutrient interactions that could confound the interpretation of nutritional status results [43,84]. Both dietary recall and biomarker data were included in this study to provide a more complete picture of intake and nutritional status. While attempts were made to adjust results for other factors that may influence the association between nutrient status and absenteeism, including demographic characteristics, there may be additional confounding factors that could introduce bias that were not adjusted for in this analysis. An investigation of the interaction between select demographic characteristics, including gender and nutrient status on absenteeism, could provide additional hypothesis-generating results.

## 5. Conclusions

A lower nutritional status of protein and several essential micronutrients, including B vitamins and antioxidants measured by dietary intake and biomarkers, are associated with more days absent from school and work due to illness/injury. The negative association may be corrected by improving dietary habits, which may include access to healthier food options, more fortified food, and dietary supplements. Results from this study highlight the importance of considering micronutrient intake from diet and supplements when assessing work and school absenteeism and developing effective interventions. The significant associations between nutritional status and absenteeism highlights the importance of surveillance of absenteeism and causes of absenteeism in future NHANES cycles. Future studies are needed to ascertain whether increasing nutrient intake in a targeted and personalized manner, particularly for nutrients not commonly found in the typical American diet or supplements, is an effective intervention to reduce absenteeism.

## Figures and Tables

**Table 1:** Population Characteristics, NHANES 2003–2008 ^a^.

	Age Group					
	6 to 18 Years			19 to 64 Years		
Demographic Characteristic	*n*	Mean	Standard Error	*n*	Mean	Standard Error
Age (years)	7429	12.0	0.10	8252	39.7	0.24
Gender (%)	7429			8252		
Males	3724	51.1	0.95	4552	53.8	0.72
Females	3705	48.9	0.95	3700	46.2	0.72
Race/ethnicity (%)	7429			8252		
Mexican American	2154	12.2	1.16	1685	8.6	0.86
Other Hispanic	422	4.3	0.83	530	3.7	0.50
Non-Hispanic white	2091	62.5	2.20	3764	70.9	1.92
Non-Hispanic black	2399	14.8	1.37	1896	11.3	1.14
Other race (including multiracial)	363	6.2	0.82	377	5.6	0.54
HH income (%)	7066			7793		
≤1.35 of PIR	2907	30.1	1.45	1948	16.8	0.85
>1.35 to <1.85 of PIR	832	9.4	0.75	818	7.9	0.41
≥1.85 of PIR	3327	60.5	1.69	5027	75.3	1.01
Education (%) ^b^	7154			7770		
<High school	2173	18.1	1.13	1651	13.2	0.72
High school	1791	26.0	1.23	1875	24.2	0.96
>High school	3190	55.9	1.41	4244	62.6	1.27
Marital status (%) ^c^	7116			8141		
Never been married	4403	69.9	1.10	4135	54.1	1.21
Divorced/Widowed	1456	18.4	1.06	1168	14.0	0.63
Currently married	1257	11.7	0.84	2838	31.9	1.22
BMI (%) ^d^	7370			8202		
Underweight	222	3.3	0.30	131	1.6	0.19
Normal	4404	63.1	1.41	2569	32.7	0.87
Overweight	1226	16.7	0.89	2720	33.1	0.75
Obese	1518	16.9	1.03	2782	32.6	1.08
Dietary supplement use (%)	7429			8252		
Yes	1663	29.4	1.26	3470	48.4	0.98
No	5766	70.6	1.26	4782	51.6	0.98

BMI, body mass index; CDC, Centers for Disease Control and Prevention; HH, head of household; NHANES, National Health and Nutrition Examination Survey; PIR, ratio of family income to poverty. ^a^ Total number of non-pregnant or breastfeeding participants ages 6 to 18 y and 19 to 64 y with valid day 1 dietary recall, valid 30-day dietary supplement use, and valid answers to number of school or work days missed due to illness or injury. ^b^ HH education level for age 6 to 18 y. ^c^ HH marital status for age 6 to 18 y. ^d^ BMI category based on percentile as specified by CDC for individuals 6 to 19 y and based on BMI cutoffs for individuals ≥ 20 y.

**Table 2:** Summary of Absenteeism Data.

	Age Group	
	Age 6 to 18 Years	Age 19 to 64 Years
*n* ^a^	7429	8252
Reported missing school or work days due to illness/injury, *n* (%) ^b^	5131 (77)	3919 (51)
Number of days missed, mean (SE) ^c^	4.00 (0.13)	4.90 (0.33)
Predicted number of missed school or work days, mean ^d^	3.99	4.79
Participants with lower absenteeism (missing days ≤ the predicted mean number of days missed), *n* (%)	5162 (64)	6930 (84)
Participants with higher absenteeism (missing days > the predicted mean number of days missed), *n* (%)	2267 (36)	1322 (16)

^a^ Total number of non-pregnant or breastfeeding participants ages 6 to 18 y and 19 to 64 y with valid day 1 dietary recall, 30-day dietary supplement use, and answers to number of school or work days missed due to illness or injury. ^b^ Refers to school days missed among 6 to 18 y and work days missed among 19 to 64 y. ^c^ Days missed (>0) over the last 12 months, including NHANES participants who did not miss any school or work days. ^d^ Based on negative binomial regression modeling.

**Table 3:** Usual Nutrient Intake from Food Alone and Food + Supplements by School Absenteeism Category ^a^.

	Food Alone				Food + Supplements			
Nutrient	Lower Absenteeism *n* = 4534		Higher Absenteeism *n* = 2002		Lower Absenteeism *n* = 4534		Higher Absenteeism *n* = 2002	
	Mean	SE	Mean	SE	Mean	SE	Mean	SE
Protein (g)	76.3	0.9	72.1 *	1.3	76.4	0.9	72.3 *	1.3
Carbohydrate (g)	286	3	286	4	286	3	286	4
Vitamin E as alpha-tocopherol (mg)	6.3	0.1	6.2	0.1	9.9	0.9	10.5	0.8
Vitamin A, RAE (μg)	598	12	588	16	765	20	833	64
Thiamin (Vitamin B1) (mg)	1.67	0.02	1.62	0.03	1.99	0.05	2.31	0.2
Riboflavin (Vitamin B2) (mg)	2.24	0.03	2.2	0.04	2.59	0.07	2.79	0.16
Niacin (mg)	23.1	0.4	22.3	0.4	25.6	0.4	25.8	0.9
Vitamin B6 (mg)	1.84	0.03	1.75 *	0.04	2.28	0.07	2.43	0.19
Folate, DFE (μg)	573	9	544 *	12	667	12	672	27
Vitamin B12 (μg)	5.53	0.1	5.13 *	0.14	6.81	0.19	6.86	0.34
Vitamin D (D2 + D3) (μg)	5.2	0.1	5	0.3	6.7	0.3	6.3	0.5
Vitamin C (mg)	82.4	2	77.6	3.1	107.6	3	105.5	4.9
Calcium (mg)	1040	16	1012	24	1068	17	1040	25
Iron (mg)	15.8	0.2	15.1 *	0.3	17.6	0.3	17.1	0.4
Magnesium (mg)	241	3	236	4	246	3	241	4
Phosphorus (mg)	1317	15	1281	23	1325	15	1289	24
Zinc (mg)	11.8	0.2	11.1 *	0.2	13.2	0.2	12.6	0.3
Copper (mg)	1.1	0.01	1.1	0.02	1.3	0.03	1.3	0.03
Selenium (μg)	101	1	96 *	2	102	1	98 *	2
Vitamin K (μg)	58.7	1.5	55.7	1.7	59.8	1.5	57.4	1.8
Total choline (mg)	266	5	248 *	6	268	5	249 *	6
Potassium (mg)	2297	33	2229	43	2301	33	2232	43
Sodium (mg)	3370	44	3275	56	3372	44	3276	56
Dietary fiber (g)	13.5	0.2	13.2	0.2	13.6	0.2	13.2	0.2
PUFA 18:3 (Octadecatrienoic) (g)	1.30	0.02	1.24	0.03	1.30	0.02	1.24	0.03
Alpha-carotene (μg)	350	40	315	38	350	40	315	38
Beta-carotene (μg)	1198	61	1109	79	1260	64	1209	84
Beta-cryptoxanthin (μg)	199	14	160 *	15	199	14	160 *	15
Lycopene (μg)	8324	425	7776	465	8331	425	7781	465
Lutein + zeaxanthin (μg)	765	29	719	33	770	29	723	32
Total fat (g)	80.7	1.1	79.1	1.1	80.7	1.1	79.2	1.1
Total saturated fatty acids (g)	28.3	0.4	27.7	0.5	28.3	0.4	27.7	0.5
Total monounsaturated fatty acids (g)	29.8	0.4	29.3	0.4	29.8	0.4	29.3	0.4
Total polyunsaturated fatty acids (g)	16	0.3	15.6	0.3	16	0.3	15.6	0.3
PUFA 20:5 (Eicosapentaenoic) (g)	0.01	0.001	0.01	0.001	0.01	0.001	0.01	0.002
PUFA 22:6 (Docosahexaenoic) (g)	0.05	0.003	0.04 *	0.004	0.05	0.003	0.04 *	0.004

BMI, body mass index; DFE, dietary folate equivalents; DRI, dietary reference intake; HH, head of household; NCI, National Cancer Institute; PUFA, polyunsaturated fatty acid; RAE, retinol activity equivalent. Lower absenteeism: with missing days ≤ the predicted mean. Higher absenteeism: with missing days > the predicted mean. * Indicates a significantly lower nutritional status associated with higher school absenteeism. ^a^ Dietary intake not available for survey 2003–2004 for total choline and 2003–2006 for vitamin D; estimated users of supplements with the specific component based on survey 2005–2008 and 2007–2008 for total choline and vitamin D, respectively. Usual intake was derived using the NCI method and the SAS^®^ macros developed by NCI for modeling of a single dietary component, and adjusting for day of recall, recall day of week (weekday/weekday), DRI, age group, gender, dietary supplement use, BMI, race/ethnicity, household income, HH education, and HH marital status.

**Table 4:** Usual Nutrient Intake from Food Alone and Food + Supplements by Work Absenteeism Category ^a^.

	Food Alone				Food + Supplements			
Nutrient	Lower Absenteeism *n* = 6086		Higher Absenteeism *n* = 1213		Lower Absenteeism *n* = 6086		Higher Absenteeism *n* = 1213	
	Mean	SE	Mean	SE	Mean	SE	Mean	SE
Protein (g)	89.8	0.9	86.3 *	1.5	89.9	0.9	86.4 *	1.5
Carbohydrate (g)	280	2	278	5	280	2	278	5
Vitamin E as alpha-tocopherol (mg)	7.8	0.1	7.4 *	0.2	32.7	2.2	34.3	3.7
Vitamin A, RAE (μg)	618	11	596	18	1244	235	946	42
Thiamin (Vitamin B1) (mg)	1.76	0.02	1.69 *	0.03	5.27	0.29	5.42	0.63
Riboflavin (Vitamin B2) (mg)	2.36	0.02	2.3	0.04	5.37	0.26	5.95	0.62
Niacin (mg)	27.1	0.3	25.7 *	0.4	38	1.4	35.2	1.5
Vitamin B6 (mg)	2.08	0.02	1.95 *	0.04	6.16	0.41	6.39	0.64
Folate, DFE (μg)	570	8	539 *	9	799	16	770	25
Vitamin B12 (μg)	5.58	0.1	5.30	0.14	27.91	2.53	34.6	10.56
Vitamin D (D2 + D3) (μg)	4.6	0.2	4.2	0.3	8.5	0.3	8.9	1.4
Vitamin C (mg)	88.0	2	77.2 *	3	192.1	11	178.8	13
Calcium (mg)	975	13	954	22	1126	17	1128	30
Iron (mg)	16.5	0.2	15.9	0.3	19.7	0.3	20.0	0.5
Magnesium (mg)	310	4	301	5	343	4	341	8
Phosphorus (mg)	1434	14	1389	25	1450	15	1403	26
Zinc (mg)	13.1	0.2	12.5	0.3	17.9	0.4	17.1	0.5
Copper (mg)	1.4	0.02	1.4	0.03	1.9	0.04	1.8	0.08
Selenium (μg)	119	1	113 *	2	134	2	126 *	3
Vitamin K (μg)	103.2	3.1	93.2 *	3.2	109.8	3.1	99.1 *	3.3
Total choline (mg)	351	4	328 *	9	353	4	329 *	9
Potassium (mg)	2824	27	2715 *	41	2842	27	2730 *	41
Sodium (mg)	3770	38	3733	62	3772	38	3735	62
Dietary fiber (g)	16.5	0.3	15.5 *	0.3	16.5	0.3	15.6 *	0.3
PUFA 18:3 (Octadecatrienoic) (g)	1.62	0.03	1.53 *	0.04	1.64	0.03	1.54 *	0.04
Alpha-carotene (μg)	788	46	629 *	61	789	47	629 *	61
Beta-carotene (μg)	1991	70	1815	95	2283	73	2049 *	111
Beta-cryptoxanthin (μg)	187	11	158	15	187	11	158	15
Lycopene (μg)	9600	371	8335 *	450	9683	371	8393 *	450
Lutein + zeaxanthin (μg)	1369	45	1255	57	1453	46	1301 *	58
Total fat (g)	89.2	1.1	88.2	1.8	89.4	1.1	88.3	1.7
Total saturated fatty acids (g)	29.6	0.4	29.6	0.6	29.6	0.4	29.7	0.6
Total monounsaturated fatty acids (g)	33.2	0.4	32.9	0.7	33.2	0.4	32.9	0.7
Total polyunsaturated fatty acids (g)	18.9	0.3	18.1	0.4	19	0.3	18.1 *	0.4
PUFA 20:5 (Eicosapentaenoic) (g)	0.04	0.003	0.04	0.003	0.05	0.003	0.04 *	0.003
PUFA 22:6 (Docosahexaenoic) (g)	0.1	0.005	0.09	0.008	0.11	0.005	0.09 *	0.008

BMI, body mass index; DFE, dietary folate equivalents; DRI, dietary reference intake; NCI, National Cancer Institute; PUFA, polyunsaturated fatty acid; RAE, retinol activity equivalent. Lower absenteeism: with missing days ≤ the predicted mean. Higher absenteeism: with missing days > the predicted mean. * Indicates a significantly lower nutritional status associated with higher workplace absenteeism. ^a^ Dietary intake not available for survey 2003–2004 for total choline and 2003–2006 for vitamin D; estimated users of supplements with the specific component based on survey 2005–2008 and 2007–2008 for total choline and vitamin D, respectively. Usual intake was derived using the NCI method and the SAS^®^ macros developed by NCI for modeling of a single dietary component, and adjusting for day of recall, recall day of week (weekday/weekday), DRI, age group, dietary supplement use, BMI, age, gender, race/ethnicity, household income, education, and marital status.

## Data Availability

The datasets analyzed during the current study are available in the NHANES repository at the following links: NHANES 2003–2004: https://wwwn.cdc.gov/nchs/nhanes/continuousnhanes/default.aspx?BeginYear=2003. NHANES 2005–2006: https://wwwn.cdc.gov/nchs/nhanes/continuousnhanes/default.aspx?BeginYear=2005. NHANES 2007–2008: https://wwwn.cdc.gov/nchs/nhanes/continuousnhanes/default.aspx?BeginYear=2007.

## Supplementary Materials

The following supporting information can be downloaded at: https://www.mdpi.com/article/10.3390/nu15204356/s1, Table S1: Usual Nutrient Intake from Food Alone and Food + Supplements Among Individuals 6 to 18 Years; Table S2: Usual Nutrient Intake from Food Alone and Food + Supplements Among Individuals 19 to 64 Years; Table S3: Distribution of the Study Population Based on Clinically Relevant Biomarker Cutpoint Levels; Table S4: Distribution of the Study Population Based on Biomarker Tertiles; Table S5: School Absenteeism and Nutrient Adequacy as Evaluated by Biomarker Levels—Adjusted Models; Table S6: Work Absenteeism and Nutrient Adequacy as Evaluated by Biomarker Levels—Adjusted Models.
